# Potential biomarkers of childhood brain tumor identified by proteomics of cerebrospinal fluid from extraventricular drainage (EVD)

**DOI:** 10.1038/s41598-020-80647-w

**Published:** 2021-01-19

**Authors:** Maurizio Bruschi, Andrea Petretto, Armando Cama, Marco Pavanello, Martina Bartolucci, Giovanni Morana, Luca Antonio Ramenghi, Maria Luisa Garré, Gian Marco Ghiggeri, Isabella Panfoli, Giovanni Candiano

**Affiliations:** 1 Laboratory of Molecular Nephrology, IRCCS Istituto Giannina Gaslini, Genoa, Italy; 2 Core Facilities-Clinical Proteomics and Metabolomics, IRCCS Istituto Giannina Gaslini, Genoa, Italy; 3 Department of Neurosurgery, IRCCS Istituto Giannina Gaslini, Genoa, Italy; 4 Unit of Neuroradiology, IRCCS Istituto Giannina Gaslini, Genoa, Italy; 5 Neonatal Intensive Care Unit, IRCCS Istituto Giannina Gaslini, Genoa, Italy; 6 Department of Neuroncology, IRCCS Istituto Giannina Gaslini, Genoa, Italy; 7 UO of Nephrology, Dialysis and Transplantation, IRCCS Istituto Giannina Gaslini, Genoa, Italy; 8 Dipartimento di Farmacia (DIFAR), Università di Genova, V.le Benedetto XV, 3, 16132 Genoa, Italy

**Keywords:** Biochemistry, Systems biology, Oncology

## Abstract

Brain tumors are the most common solid tumors in childhood. There is the need for biomarkers of residual disease, therapy response and recurrence. Cerebrospinal fluid (CSF) is a source of brain tumor biomarkers. We analyzed the proteome of waste CSF from extraventricular drainage (EVD) from 29 children bearing different brain tumors and 17 controls needing EVD insertion for unrelated causes. 1598 and 1526 proteins were identified by liquid chromatography-coupled tandem mass spectrometry proteomics in CSF control and brain tumor patients, respectively, 263 and 191 proteins being exclusive of either condition. Bioinformatic analysis revealed promising protein biomarkers for the discrimination between control and tumor (TATA-binding protein-associated factor 15 and S100 protein B). Moreover, Thymosin beta-4 (TMSB4X) and CD109, and 14.3.3 and HSP90 alpha could discriminate among other brain tumors and low-grade gliomas plus glyoneuronal tumors/pilocytic astrocytoma, or embryonal tumors/medulloblastoma. Biomarkers were validated by ELISA assay. Our method was able to distinguish among brain tumor vs non-tumor/hemorrhagic conditions (controls) and to differentiate two large classes of brain tumors. Further prospective studies may assess whether the biomarkers proposed by our discovery approach can be identified in other bodily fluids, therefore less invasively, and are useful to guide therapy and predict recurrences.

## Introduction

Pediatric brain tumors are a leading cause of tumor-related mortality in children^1,2^. Although diagnostic procedures vary according to the tumor location and estimated stage, diagnosis of CNS tumors relies on histopathological analysis and neuroimaging^2,3^. The technological advances in neuroimaging, neurosurgery, oncology, and radiotherapy improved survival of childhood CNS tumors^3,4^. Neuronavigation and endoscopy are revolutionizing pediatric neuro-oncology^5^. However, there is the need to identify biomarkers, i.e. measurable substances reflecting the presence of a tumor, to facilitate diagnosis, therapeutic stratification, and detection of residual disease or recurrence^3,6^. Among the multiple potential pitfalls in the decision-making in the case of brain tumours, there is difficulty in differentiating between true tumor progression or recurrence versus, for example radiation reactions^7^. In fact, radiotherapy after surgery is the most common treatment option for many brain tumors^1^.

New insights in child brain tumors are coming from large-scale genomics profiling or proteomic studies, especially from high-throughput technologies^8–11^. The search for biomarker has exploited novel targets: for example, the use of exosomes in the sera of glioblastoma patients^12^. However, serum presents a major hindrance to the finding of tumor-specific protein markers due to the selectivity of the blood–brain barrier^13^. By contrast, cerebrospinal fluid (CSF) is considered a promising source for pediatric CNS tumor biomarker discovery^14^. Most of CSF is produced by the choroid plexus and the rest originate from drainage of interstitial fluid from the CNS^15^. CSF is accessible, in contact with both brain tissue and tumor bulk, also being a primary route for metastases. It has been shown to contain many unique proteins^10,14,16–18^. In a previous mass spectrometry (MS) comprehensive characterization of healthy normal CSF samples by lumbar puncture after immunoaffinity separation 2630 proteins were identified in non-tumor subjects, half of which were CSF-specific, to represent a comparative standard^16^. A panel of urinary biomarkers was reported showing significant elevations of MMP-2, MMP-9, MMP-9/NGAL which correlate with presence of disease in brain tumor patients, compared with controls^19^.

A few MS proteomic studies of child brain tumor-associated CSF have been reported^18,20,21^. Pediatric primitive neuroectodermal tumors and ependymomas were studied by a proteome-wide approach, and three proteins (stathmin, annexin A1, and calcyphosine) were identified as tumor-specific^20^. Recently, Spreafico et al.^18^ characterized the CSF proteome of children bearing CNS tumors, to identify biomarkers predictive of metastatic spread. Out of a number of low abundant proteins identified, six (type 1 collagen, insulin-like growth factor binding protein-4, procollagen C-endopeptidase enhancer 1, glial cell-line derived neurotrophic factor receptor α2, inter-alpha-trypsin inhibitor heavy chain-4, neural proliferation and differentiation control protein-1) were selected as potential biomarkers of metastatic spread. Another study analyzed endogenous peptides extracted from CSF was recently conducted by LC-MALDI MS and it was found that these originated from a number of proteins involved in different disorders of the central nervous system^14^.

Here we conducted a proteomic and bioinformatic analysis of CSF samples collected from the extra ventricular drainage (EVD) of 29 consecutive patients treated for common brain tumor types including both malignant and benign histopathologies, and compared these to CSF samples sourced from the EVD of 17 non-tumor patients and identified some putative protein biomarkers. Aim of our study was to utilize the considerable volume of CSF from EVD routinely treated as a waste, to seek for predictive protein biomarkers for estimating probability of a tumoral condition from any other needing an EVD, although characterized by pathological CSF production (controls). Also, we aimed to assess whether any of the putative biomarkers could discern specific brain tumor types.

## Results

### Protein composition

**Figure 1:**
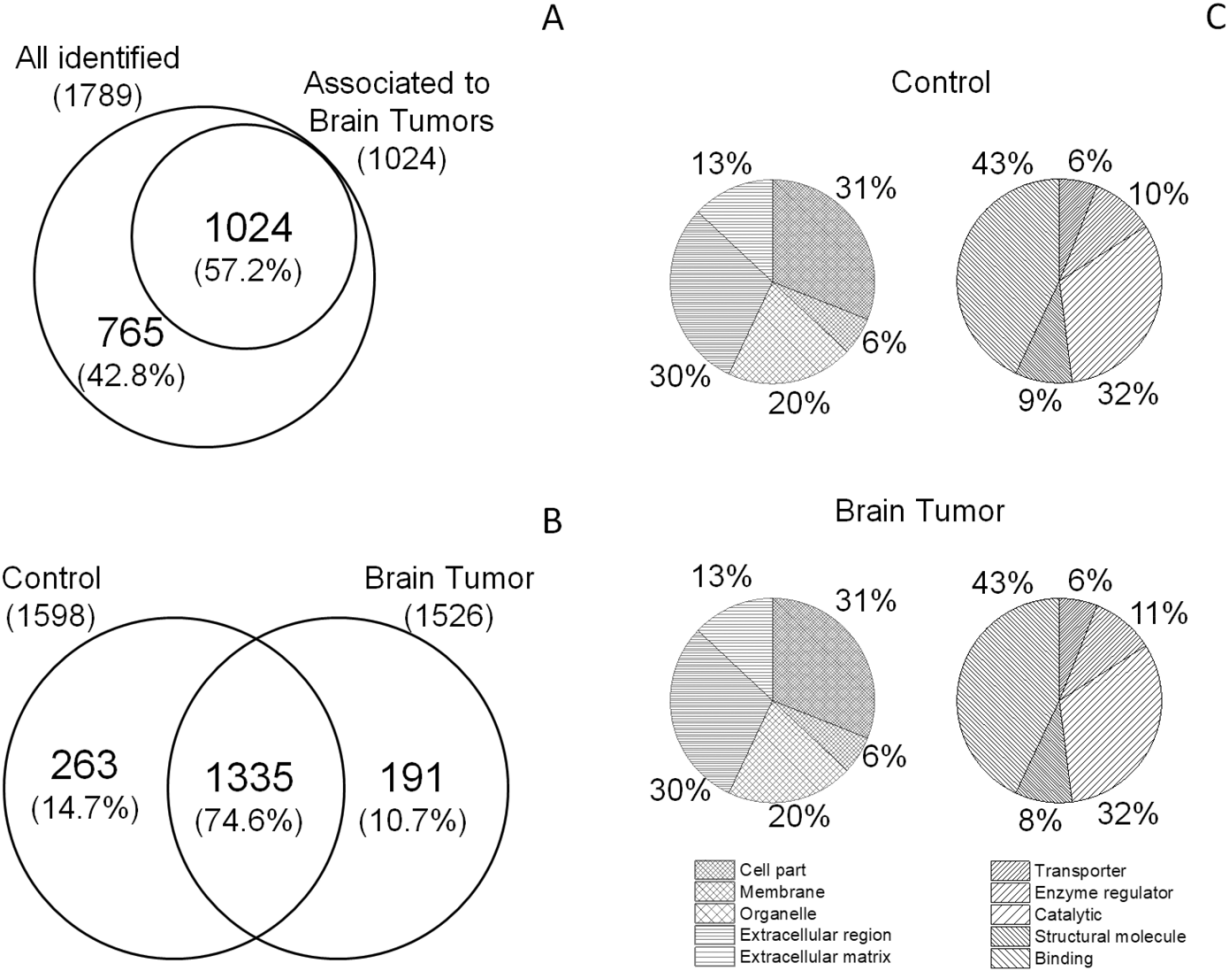
Venn diagram and pie chart of the identified proteins. Venn diagram of proteins previously described as associated to brain tumors (**A**) and total proteins identified in CSF from EVD of control and brain tumor samples (**B**). Both diagrams show common and exclusive proteins. Numbers represent the distinct proteins. (**C**) Pie chart of the enrichment of gene ontology annotation terms in the two groups.

We analyzed the proteome of the CSF from EVD from 29 children bearing different brain tumor types and 17 controls. One sample was withdrawn from each patient. 1789 proteins were identified (Supporting Table S1), 1024 (57.2%) of which have been previously described in brain tumor (www.uniprot.org)^22^ (Fig. 1A). Out of 1789, 1598 and 1526 proteins were identified in non-tumor or tumor brain samples, respectively. In particular, 1335 proteins (74.6%) out of the total overlapped, while only 263 (14.7%) and 191 (10.7%) were exclusive of either condition (Fig. 1B). Moreover, stratifying brain tumor samples according to three clinical groups (i.e. low-grade gliomas (LGG) plus glioneuronal tumors (GT), embryonal tumors (EMB), Other Brain Tumors), it was observed that 870 proteins (48.6%) were overall overlapping, while 30 (1.7%), 115 (6.4%) and 12 (0.7%) were exclusive for LGG plus GT, EMB, and Other Brain Tumors, respectively.

The identified Proteins were classified according to gene ontology (GO) signatures, on the bases of cellular component (CC) and molecular function (MF). Proteins displayed the same percent CC subdivision, being 31% cellular, 30% extracellular, 20% organelle, 13% extracellular matrix and 6% membrane. MF classification was also very similar for the control and tumor groups (43/43% binding activity; 32/32% catalytic activity, 10/11% enzyme regulator activity, 9/8% structural molecule and 6/6% transporter activity) (Fig. 1C).

**Figure 2:**
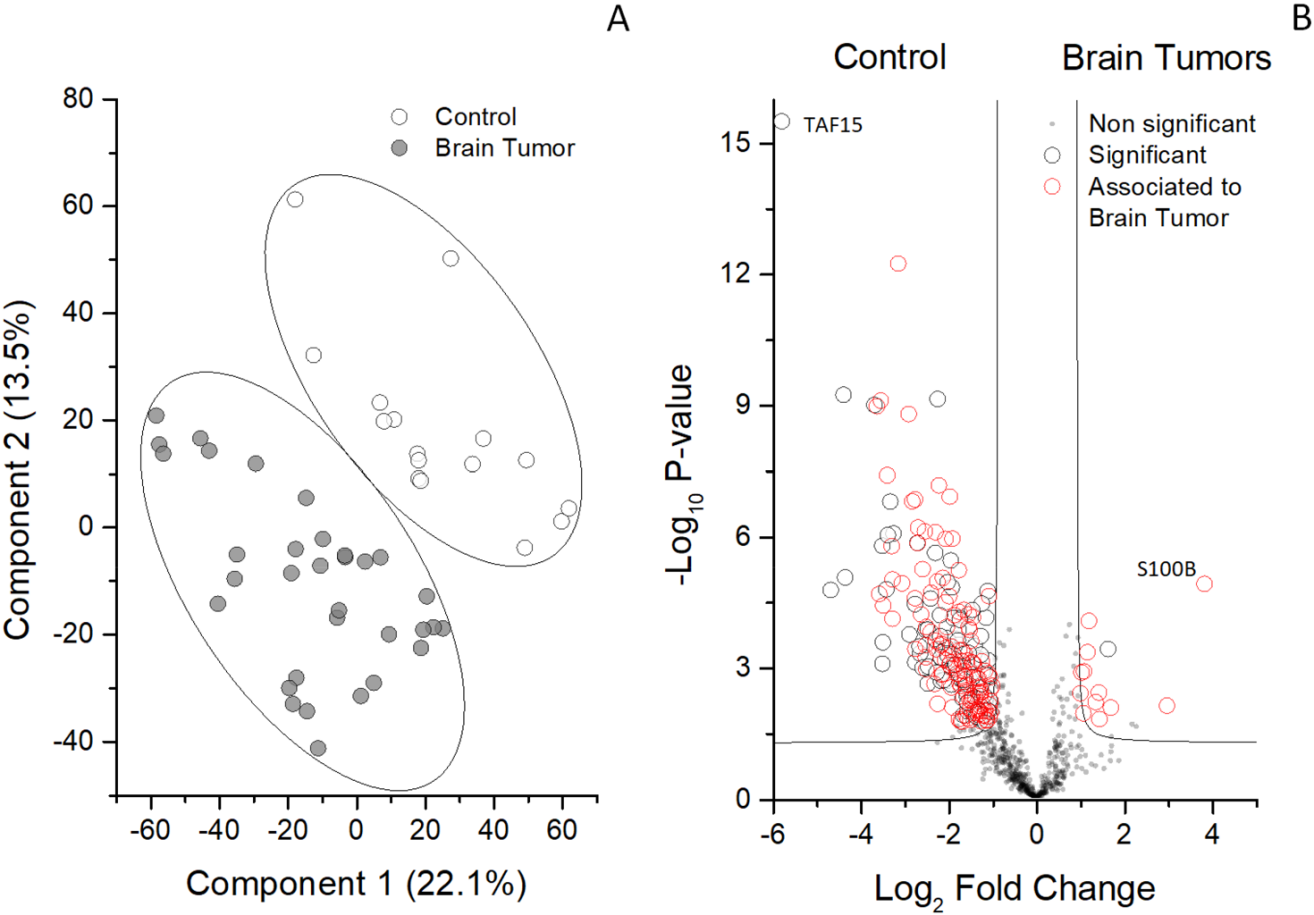
Multidimensional scaling (MDS) and volcano plot of CSF proteome from EVD of control and brain tumor samples. (**A**) Scatter plot of MDS analysis of control (white circles) and brain tumor samples (grey circles). Ellipsis indicate 95% confidence interval. Plot shows clustering of two distinct groups (tumor and control samples). (**B**) Volcano plot of all the identified proteins in all samples. Grey, open black and red circles indicate the non-significant, significant or previously described as associated to brain tumor protein changes between the two groups, respectively. Black line indicates the limits of statistical significance. Grey circles above the black line indicate the proteins with an identity < 70%.

**Figure 3:**
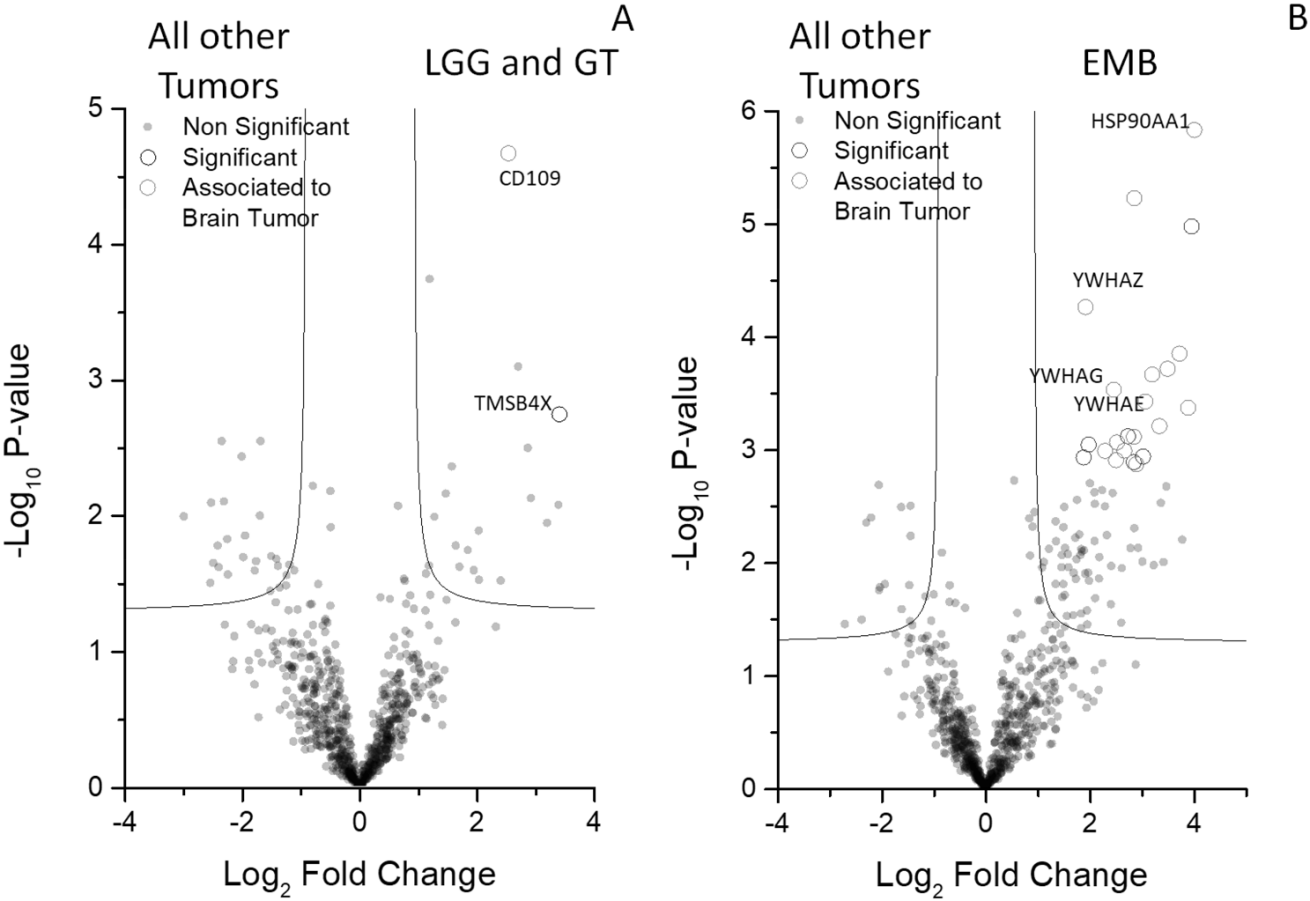
Volcano plots of CSF proteome from EVD of LGG and GT and EMB compared with all other classes of brain tumor. Volcano plot for (**A**) LGG and GT or (**B**) EMB compared with all other brain tumor samples. Grey, open black and red circles indicate the changes for the non-significant, significant or previously described as associated to brain tumor proteins among the two groups, respectively. Black line indicates the limits of statistically significant. Grey circles above the black line indicate the proteins with an identity < 70%.

Despite high identity overlapping, discrimination between control and tumor samples was good (Fig. 2A). Differences between the two conditions were analysed by T test and machine learning systems. A total of 241 proteins was highlighted, 228 and 13 of which were enriched in control and tumor, respectively (Fig. 2B). Out of the 241 statistically significant proteins, 141 proteins have been previously described in brain tumor^22^. Besides, comparing all other brain tumor types to LGG plus GT (Fig. 3A), or to EMB tumors (Fig. 3B), we identified 2 and 22 proteins statistically up-regulated in either clinical groups respectively, one (1/2) and 16 (16/22) of which have been previously described in brain tumor^22^ (Supporting Table S2).

Considering the suitable homogeneity and size of pilocytic astrocytoma (PA) and medulloblastoma (MB) samples in the LGG plus GT and EMB groups, respectively, we compared PA and MB to all other brain tumors, to assess whether any biomarker could discern either type of tumour. 12 and 70 proteins were significantly changed in PA or MB respectively (Supporting Table S2). 6 out of the 12 and 10 out of the 70 significantly changed proteins were previously described in PA and MB, respectively^22^.

**Figure 4:**
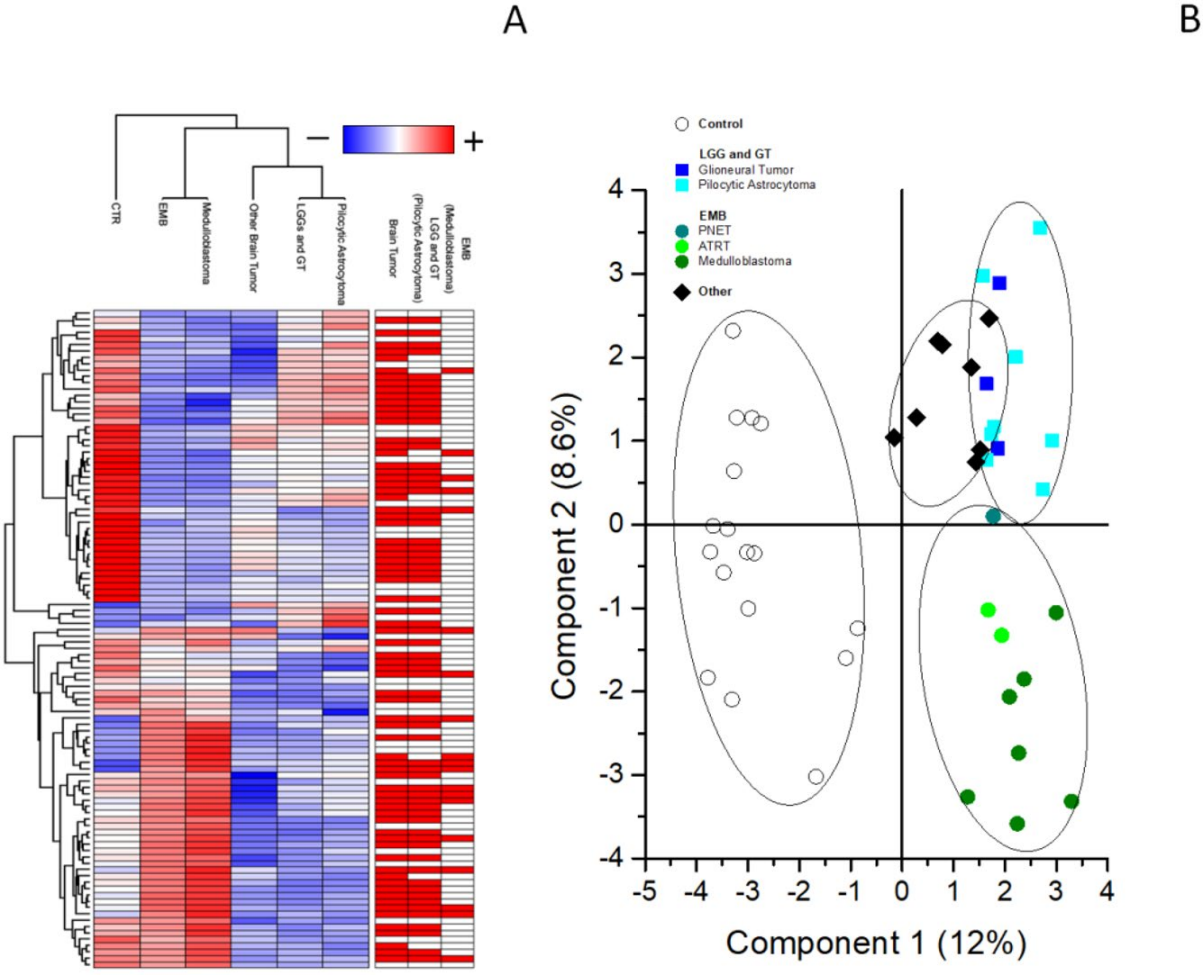
Heatmap and PLS-DA of highlighted proteins. (**A**) Heatmap of the proteome profile of 104 proteins highlighted by the statistical analysis (see Supplementary Table S3 for details). Each row represents a protein and each column a clinical group. Normalized Z scores of protein abundance are depicted using a pseudocolor scale (red, white and blue indicating positive equal and negative expression, respectively) compared to each protein value. The dendrogram displays unsupervised hierarchical clustering analysis. Similar sample/proteome-profile values are next to each other. (**B**) Scatter plot of PLS-DAS analysis of proteome profile of control (white circles), LGG and GT (blue and cyan squares), EMB (light and dark green circles) and other mixed brain tumors (black diamonds). Symbols and ellipsis indicate each sample and the 95% confidence interval of the four clusters. These proteins can clearly discriminate between the different conditions.

To better describe the differences among the control and the three different brain tumor clinical groups, ANOVA test, PLS-DA and SVM learning analyses were performed. ANOVA test highlighted 302 proteins (Supporting Table S2). Then, the priority of the 302 proteins was established by means of SVM learning and PLS-DA analyses, to distinguish among control and the three clinical groups. Priority was determined using the rank list and the variable importance in projection (VIP) score obtained using SVM and PLS-DA, respectively. Both analyses identified the same protein priority. Moreover, the combined use of statistical analyses and machine learning revealed a ranked core panel of 104 proteins maximizing the discrimination among control and tumor conditions and between the whole tumor group and each clinical group (Supporting Table S3). The expression profile of this core panel of proteins, after Z-score normalization, is visualized in the heatmap shown in Fig. 4A. The k-means analysis associated to PLS-DA showed the presence of four different clusters, corresponding to the four conditions, with a clear discrimination between control and tumor and a good discrimination between the three tumor clinical groups (Fig. 4B). No further subdivision resulted statistically significant within each brain tumor clinical group, with this combined approach.

The complex of combined analyses identified six 6 potential biomarkers able to distinguish from brain tumor from control and to stratify the former into three different clinical groups. In particular, TAF15 and S100B resulted the most promising biomarkers for the discrimination between control and tumor (Fig. 2B). Also, the most promising biomarkers for the discrimination between LGG plus GT and PA were TMSB4X and CD109 (Fig. 3A and Supporting Figure S1A), while the most promising biomarkers for the discrimination between EMB MB and all the other brain tumors were 14.3.3 (YWHA-Z,G,E) and HSP90 alpha (Fig. 3B; and Supporting Figure S1B).

The considerable diversity in the expression profile of the proteins identified in control and tumor samples may imply their different roles. To assess this, we performed GO enrichment analysis based on annotation extracted from various databases. A network diagram of biological processes summarizes the results (Supporting Figure S2). Processes were clustered in four groups in function of their GO annotation. In the brain tumor cluster, a down-regulation of proteins involved in cell–cell and cell–matrix adhesion, cytoskeleton stability, cellular differentiation and leukocyte uptake was found. By contrast, proteins involved in matrix remodeling, production and regulation of cytokines were upregulated in the control group.

### ELISA data verification

**Figure 5:**
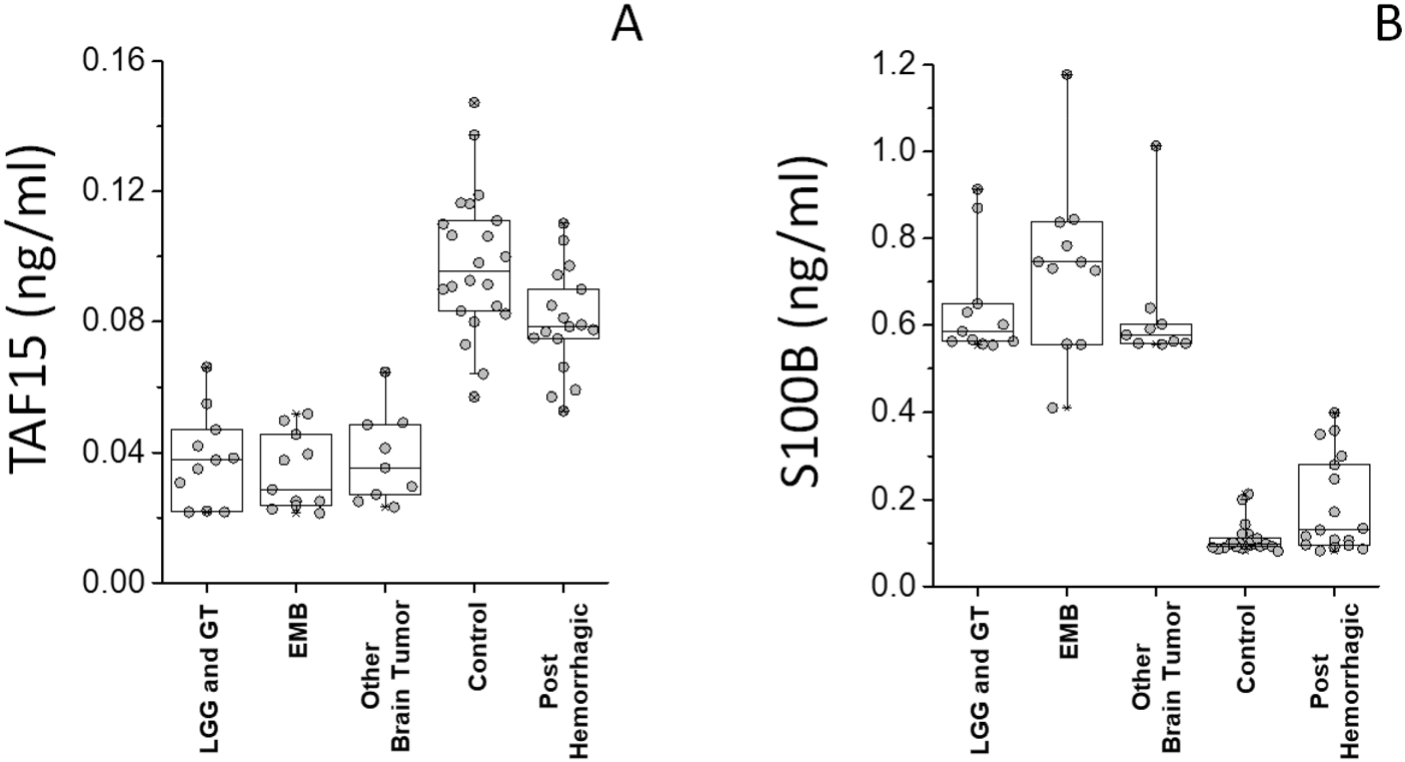
TAF and S100B ELISA assay. Box plots showing the median and interquartile range value for (**A**) TAF15 and (**B**) S100B CSF proteins in all subjects.

**Figure 6:**
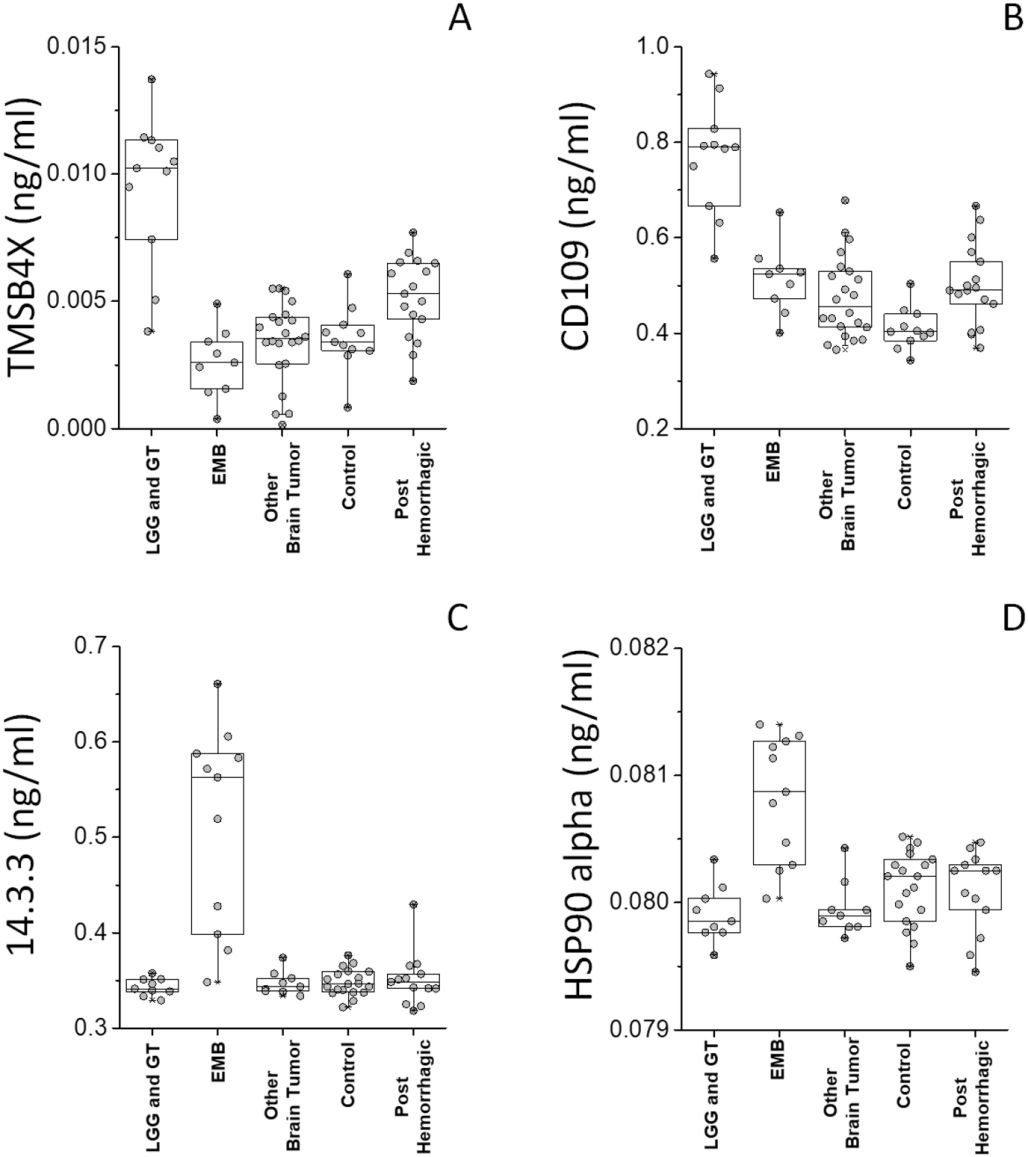
TMSB4X, CD109, 14.3.3 and HSP90 alpha ELISA assay. Box plots show the median and interquartile range value for (**A**) TMSB4X, (**B**) CD109, (**C**) 14.3.3 and (**D**) HSP90 alpha from CSF from EVD in all subjects. TMSB4X and CD109, or 14.3.3 and HSP90 alpha were statistically more abundant in LGG and GT or EMB, respectively, compared to all other groups.

Commercial ELISA Kits were used to determine the levels of the selected biomarkers in 68 CSF samples, distinct in 37 non-tumor samples (out of which 15 were post hemorrhagic and 22 were congenital hydrocepahlus) and 31 brain tumor samples (out of which 11 belonged to the LGG plus GT, 11 to the EMB and 9 to the Other Brain Tumor clinical groups, respectively). TAF15 was statistically more abundant in non-tumor samples, as compared to the tumor group. S100B showed the opposite profile (Fig. 5). TMSB4X and CD109, or 14.3.3 and HSP90 alpha were respectively statistically more abundant in LGG plus GT or EMB clinical groups (Fig. 6). The median/IQR, of the selected potential biomarker in each group are reported in Table 1, also reporting the cutoff, likelihood ratio, area under the curve (AUC), their confidence interval (CI) and p-value of each ROC curve. Supporting Figure S3 shows the AUC of each ROC analysis, resulting > 0.9 in all cases, therefore classified as excellent.

**Table 1:** ELISA dosage.

	TAF15	S100B	TMSB4X	CD109	14.3.3	HSP90 alpha
Control	0.09 (0.08–0.11)	0.1 (0.09–0.11)	0.003 (0.003–0.004)	0.43 (0.39–0.53)	0.34 (0.34–0.36)	0.08 (0.08–0.08)
Post hemorragic	0.08 (0.07–0.08)	0.11 (0.09–0.13)	0.01 (0.004–0.01)	0.48 (0.41–0.51)	0.35 (0.34–0.36)	0.08 (0.08–0.08)
LGG and GT	0.03 (0.02–0.04)	0.57 (0.56–0.6)	0.01 (0.01–0.01)	0.79 (0.67–0.83)	0.34 (0.34–0.35)	0.08 (0.08–0.08)
EMB	0.03 (0.02–0.04)	0.75 (0.64–0.81)	0.003 (0.003–0)	0.4 (0.39–0.43)	0.56 (0.41–0.59)	0.08 (0.08–0.08)
Other Brain Tumor	0.04 (0.03–0.05)	0.58 (0.56–0.6)	0.003 (0.002–0.003)	0.52 (0.47–0.54)	0.34 (0.34–0.35)	0.08 (0.08–0.08)
Kruskall-Wallis Test (P-value)	< 0.001	< 0.001	< 0.001	< 0.001	< 0.001	< 0.001
ROC analysis						
No Brain Tumors vs Brain Tumors AUC (CI); P-value	0.98 (0.97–1);P < 0.0001	1 (0.99–1); P < 0.0001	–	–	–	–
LGG and GT vs All other Brain Tumors AUC (CI); P-value	–	–	0.99 (0.92–1); P < 0.0001	0.97 (0.95–1); P < 0.0001	–	–
EMB vs All other Brain Tumors AUC (CI); P-value	–	–	–	–	0.97 (0.89–1); P < 0.0001	0.96 (0.9–1); P < 0.0001
CV%	3.6	4.8	4.4	3.8	3.1	2.9
Lower limit of quantification	0.01	0.04	0.0001	0.15	0.15	0.04
Cut-off	0.06	0.30	0.005	0.59	0.36	0.08
Sensitivity %	84 (67–93)	100 (88–100)	89 (52–100)	89 (52–100)	91 (59–100)	73 (40–94)
Specificity %	96 (82–100)	97 (84–100)	95 (75–100)	95 (75–100)	94 (73–100)	94 (73–100)
Likelihood ratio	24	32	18	18	16	13

ELISA dosage was conducted on the core panel of potential biomarkers of cerebrospinal fluid from extraventricular drainage. Their receiver operating characteristic (ROC) curve analysis, for the different comparisons reported in this study was performed. Biomarker dosage is expressed in ng/ml as median and interquartile range. In the ROC analysis AUC, 95% of the confidence interval (CI), P value, cut-off, sensitivity, specificity and likelihood ratio are reported.

## Discussion

Proteomic and transcriptomic data represent promising tools for the understanding of the paediatric brain tumor and natural history^9,23^. The goal of the present systems biology preliminary discovery study was to find novel biomarkers of brain tumor and to assess whether any of those could distinguish single tumor types.

Data show that the putative protein biomarkers here identified from child CSF, verified by ELISA assay, are able to discriminate brain tumor from non-tumor samples and discern two tumor types (PA and MB) out of all tumors. ELISA data correlate with MS data, however with a lower fold change, as MS displays a wider dynamic range.

TAF15 and S100B, were the best biomarkers to differentiate the tumor from the non-tumor conditions. Tumoral condition seems discriminative per se. Human TATA-box binding proteins are RNA-binding multifunctional proteins belonging to the FET family, that function in splicing and mRNA transport, and possess a potent transcriptional activation domain^24^. TAF15 is associated with RNA polymerase II, playing a role in role in subcellular targeting of translation^24,25^. FET proteins have been considered proto-oncogenic as they form oncogenic fusion genes and proteins. Consistently, it was reported that TAF15 levels decrease during differentiation, TAF15 knockdown negatively affecting cell proliferation^24^. Conversely high TAF15 levels post-transcriptionally regulate cell cycle and are pivotal for rapid cellular proliferation^24^. TAF15 is expressed in a number of cancers, especially sarcomas^25^, although not specifically associated to brain tumor. An oncogenic from of TAF15 fused with the transcription factor CIZ/NMP4, has been associated to acute leukemia^26^. Here, TAF15 was statistically more abundant in non-tumor group. Notably, the ability to stabilize mRNA is not necessarily oncogenic, in fact recently it was found that TAF15 was downregulated in glioma cells^27^. Overexpressed TAF15 stabilized long intergenic non-protein coding RNA 665, inhibiting the malignancy of glioma cells. Such key role of TAF15 the behaviour of glioma cells, appears in line with our results.

S100B is a glial-specific calcium-binding protein involved in cell cycle regulation^28^. It is considered a clinical marker of glial activation and brain damage^28^ with a potential in monitoring efficacy of treatment^12^. We found S100B upregulated in the brain tumor group, consistently with its role as neuronal survival protein. S100B has been described as a biomarker in other brain related pathologies such as traumatic brain injury, damaged blood–brain barrier^29^ or CNS infection^30^. CSF S100B is specific of cerebral parenchyma injury and when elevated is a diagnostic biomarker for CNS infection and a predictor of unfavorable outcome in infectious encephalitis^30^. Serum S100B levels have a prognostic value for survival in adult patients with recurrent, but not newly diagnosed glioma^31^, however there is no evidence for S100B to be specific for child brain tumor.

Samples from two tumor types (PA and MB) possessed the appropriate size and homogeneity allowing to investigate whether the biomarkers associated to them could discriminate each of these from all the other tumors. TMSB4X and CD109 were also associated to tumor conditions, being as well able to discriminate PA from all other tumors. Most gliomas in children are slow-growing lesions (LGG), classified as grade I or II by the WHO classification of CNS tumors^32^. By contrast, pediatric High Grade Gliomas (pHGGs, WHO grade III or IV), are a heterogeneous group of rapidly progressing tumors mainly containing an astrocytic component. Notwithstanding the aggressive radiation therapy treatment protocol available^33^, pHGGs remain largely incurable. GT are extremely rare childhood mixed neuronal-glial tumors. Most of them are temporal lobe slow growing grade I tumors^34^. TMSB10 has been proposed as a serum biomarker and a potential therapeutic target in breast tumor^35^. TMSB4X is a cytoskeletal protein inhibiting actin polymerization, involved in tumorigenesis. It is highly expressed in certain tumor cells, including NSCLC lung tumor, where it has been proposed as a molecular target for therapy^36^. TMSB4 is also a positive regulator of ATP biosynthetic processes. It was shown that TMSB4 binds to the β subunit of F_1_F_o_-ATP synthase increasing cell surface ATP levels, in turn triggering an extracellular pathway involving P2X4 ATP receptors, inducing cell migration^37^.

CD109 is a glycosylphosphatidylinositol-anchored cell surface antigen expressed by T-cells and endothelial cells. CD109 is already considered a marker of the glioma cells that populate the perivascular tumor, promoting its progression by suppressing TGF-β signalling^38^. CD109 is upregulated in various tumour cell lines including Glioblastoma (GBM)^39^. GBM cancer stem cells expressing CD109 would be involved in the progression from low-grade to high-grade glioma^40^.

Among the proteins found significantly associated to PA (Supplemental Table S2), the tripartite motif-containing protein 33 (TRIM33), is a transcriptional corepressor suppressor of brain tumor development^41^. PA is the most frequent primary, relatively benign (WHO grade I) child brain tumor. It is essential to distinguish PA from the more aggressive diffuse gliomas^42^.

HSP90 alpha and 14.3.3 appear promising biomarkers able to discriminate between EMB/MB, originating from embryonic brain cells^43^, from all other brain tumors (Supporting Figure S1B). MB is the most common malignant brain tumor in children^2^, categorized into four distinct variants^32,43^. As an inhibitor of apoptosis, 14.3.3 promotes tumor survival and chemoresistance, and has been proposed as a novel molecular target for tumor therapy^44^. Heat shock proteins (HSPs) are a superfamily of chaperones overexpressed in a number of tumors, among which MB^45^.

Interestingly, proteins involved in cell–cell and cell–matrix adhesion and cytoskeletal stability^46^ were generally down-regulated in brain tumors. On the other hand, matrix remodeling and cytokine control proteins were upregulated in brain tumor, consistently with acquisition of invasivity^47^.

The six putative biomarkers here identified (TAF15, S100B, TMSB4X, CD109, HSP90, 14.3.3), a few of which have been previously described in childhood brain tumor^22^, appear more specific than those reported in previous similar studies^18,48^. These, after validation, can be searched for in other body fluids, where hopefully they can be less invasively identified, as the next step of their clinical implementation.

In search for biomarkers for diagnosis and risk stratification, CSF appears to be an appropriate medium for informative liquid biopsies^46^. A major drawback of using CSF for sampling and searching for biomarkers is the invasive nature of obtaining CSF samples, either via lumbar puncture or EVD, the latter being even more invasive. Here we collected CSF from EVD, a source potentially available for serial sampling without volume restraints or further ethical problems. In fact, withdrawal of CSF from EVD does not add any invasive procedure to those subjects who carry an EVD. Also, the availability of an adequate amount of CSF^9^ may be important to find low abundance proteins. Of course, the composition of CSF from EVD is far from the physiological one, due to the high volumes produced per day, and the absence of physiological modifications, such as mixing due to heartbeat, respiration and posture^49^.

In this respect, our conditions are different from the study set to identify biomarkers predictive of metastatic spread^18^ where CSF samples were collected by lumbar puncture, the controls being children with extra-CNS non Hodgkin lymphoma. This study is also different from the proteomic analysis of CSF from 10 children with diffuse intrinsic pontine glioma (DIPG) versus 4 controls^48^, that identified 528 unique proteins. Upregulation of Cyclophillin A, also detected in urine and serum, and dimethylarginase 1 was found^48^.

Our controls were children needing EVD insertion for causes unrelated to tumor, including congenital hydrocephalus and hemorrage. This also allowed us to cluster hemorrhagic patients versus tumor conditions. Hemorrhage and blood in CSF may seem to impact the comparison, in that many inflammatory and plasma proteins were found, which was functional to verifying whether any condition affecting the brain and needing and EVD can be differentiated on a biomarker basis from the tumoral condition, therefore it represents a control. The identification of plasma and inflammatory proteins increased variability of non-tumor sample, therefore the validity of the proteins identified in the tumor group.

A limitation of our study is the different age of control group (encompassing post hemorrhagic and congenital hydrocepahlus patients), versus tumor group. Indeed, such difference was quite inevitable, due to the younger age of congenital hydrocephalus patients. Nonetheless, the median age value of the controls was 12 months, while for the control group it ranged among 4 and 10 years (Table 2). Therefore, the control group does not exclusively include one extreme of life. Most variability in CSF composition is in fact seen at the two extremes of life. Qualitative variability exists in the new-born (below 30 days from birth)^50^. In the elderly there is an increase in CSF volume due to diminished brain volume, and an increase in CSF glucose concentrations^51^. Apart from quantitative differences (daily production ranges between 25 ml in the newborn and around 500 ml in adults^52^). Good clinical practice considers CSF of individuals over 3 years of age equal to the adult from the point of view of overall variability of its most important parameters. No difference was found between a child of 6 months and another of 12 years of age as revealed by a study on the age-dependent reference values for CSF protein in children^53^. Nonetheless, these considerations appear not relevant here, as we have utilized a non-physiologic largely overproduced CSF. On the other hand, influence of sex is irrelevant, as children were all prepuberal, moreover some of CSF parameters are never related to gender^51^.

**Table 2:** Clinical characteristics of the sixty-eight patients enrolled in the study.

Groups	MS / ELISA	Sex (F/M)	Age (year)
**Control (37)**			
Congenital hydrocephalus (22)	17/22	13/9	6 (4–10)
Post-hemorrhagic (15)	0/15	4/11	6 (4–10)
**Low-grade gliomas and glioneural tumors (11)**			
Pilocyticastrocytoma (8)	8/8	4/4	8 (3–15)
Gangliocytoma/Ganglioglioma (3)	3/3	2/1	9 (5–11)
**Embryonaltumors (11)**			
Medulloblastoma (7)	7/7	4/3	5 (0–15)
Atypical teratoid rhabdoid tumor (ATRT) (3)	2/3	2/1	1 (1–2)
Primitive neuroectodermal tumor (1)	1/1	0/1	7
**Other (9)**			
Meningiomas (2), germ cell tumors (2), ependymomas (2), plexus papillomas (2), hemangioblastoma (1)	8/9	3/6	9 (0–15)

All patients with Brain tumors had a histological diagnosis. The abbreviations MS and ELISA correspond to samples (i.e. patients) analysed by mass spectrometry (46) and/or ELISA assay (68), respectively. The total number of patients in each clinical group is reported in brackets. Age is reported as years (median and range).

Our data are consistent with the hypothesis that the CSF proteome reflects the brain tumor microenvironment^54,55^ and can be a source of biomarkers. The CSF protein signature can detect early stage brain tumors in animal models^56^. CSF is also considered a primary route for metastases^57^. Despite having extensively been studied for the detection of tumor biomarkers, few of CSF markers have found a clinical application^11^. While there is consensus about the fact that CSF is amenable for the research of biomarkers of neurological disease, literature reports high variability across studies that hinders implementation in clinical practice^58^. Notwithstanding the fforts in identifying novel CSF biomarkers, the high variability observed across different studies has hampered their clinical implementation. Such variability is partly due to protein stability issues, highlighting the importance to standardize withdrawal procedures^58^. Optimization of protocols for CSF sample collection and treatment have been recently proposed for biomarker studies, although from lumbar puncture^59^. There is also the issue CSF access to tumor site: CSF is considered an extension of the CNS extracellular compartment. Tumor cells are in turn inextricably linked to their microenvironment. Therefore, tumor-related markers can be more concentrated near the tumor. Moreover, CSF is modified on its passage from the ventricles to the lumbar sac also depending on its flow rate^60^. Moreover, approximately 80% of the total CSF protein is derived from the plasma^61^, and those derived from the brain parenchyma account for only a low percentage. In this respect, the difference between CSF obtained from the lumbar cistern versus that obtained from the ventricular CSF may have allowed us to identify novel putative biomarker proteins because of the characteristics of our sample.

A “normal” (or healthy) ventricular or lumbar CSF is not obtainable for ethical reasons, thus our study design is the next best. The implications of this are that we ruled out the issue that many studies comparing CSF from lumbar puncture encounter of finding a proper control sample.

It is tempting to presume that the biomarkers here proposed represent a synergic panel to be exploited in the treatment follow-up and identification of recurrences. In fact, as many new treatment options are available for child brain tumor, the interpretation of post-treatment imaging is becoming parallelly more complex. Presently, response to treatment and identification of recurrence essentially rely on advanced MRI, including diffusion-weighted (DWI)^7^ and proton magnetic resonance spectroscopic (MRS) metabolite profile imaging^62^. However, to effectively distinguish between recurrence and radiation necrosis, a long-term complication of radiation treatment^7^, MRI can be insufficient^63^.

Future studies can translate the results of the present study into clinical application. It would allow to distinguish among two groups of tumors (pilocytic astrocytoma and medulloblastoma) compared with all other classes of brain tumor, and each of these from a cohort of both non-tumor and hemorrhagic subjects. It will also be useful to investigate whether the biomarkers here proposed can be detected with the same statistical efficiency in other biofluids, for example serum or urine, for non-invasive clinical use.

## Materials and methods

### Sample collection and patient information

All consecutive pediatric patients with a brain tumor admitted to the Neurosurgery Unit of Giannina Gaslini Children’s Hospital in the period 2015–19, who required placement of an EVD catheter were eligible for inclusion in the study. CSF samples otherwise destined to waste were collected not further invasively at the first change of the disposable bag of EVD by a sterile procedure (no seriate sampling was performed), after ethical approval and informed consent signed by the children’s parents/guardians. Control samples were obtained from patients with congenital hydrocephalus (grades III to V) unrelated to a brain tumor, who underwent ventriculostomy and EVD insertion (control groups in mass spectrometry experiment) and post-hemorrhagic (used in addition of previous groups for ELISA verification). The clinical data are reported in Table 2. All patients treated for a brain tumor had histological diagnosis centrally reviewed and performed according to the World Health Organization (WHO) classification^32^.

In particular, mass spectrometry discovery approach was done on 46 CSF EVD samples (one from each of the 46 patients), stratified in 17 congenital hydrocephalus (control), 11 low-grade Gliomas and Glioneural tumors (LGG and GT), 10 embryonal tumors (EMB), 8 other brain tumor samples. By contrast, ELISA verification of the mass spectrometry results was performed on 68 patients/samples, i.e. the previous 46 samples with the addition of 22 congenital hydrocephalus (controls), plus 15 post-hemorrhagic, 11 low-grade Gliomas and Glioneural tumors (LGG and GT), 11 embryonal tumors (EMB), 9 other brain tumor samples (see Table 2 for detail). All CSF EVD samples were centrifuged at 3000*g* for 10 min, to remove cells and debris and immediately frozen at − 80 °C until use.

### Sample preparation for mass spectrometry (MS) and mass spectrometer setup

According to Bruschi et al.^64^ sample pellets obtained by sodium deoxycholate and trichloroacetic acid precipitation were lysed, reduced and alkylated in 100 μl 6 M Guanidine, 10 mM TCEP, 4 mM CAA, 100 mM Tris pH 8.5, and the protein concentration measured using a tryptophan assay^65^. Then, 25 μg of each sample were digested by adding trypsin and LysC (at a 1:50 and 1:100 ratio of enzyme to sample protein respectively, both in micrograms), mixing and incubating at 37 °C overnight. Digested samples were loaded onto StageTips^66^. Resulting peptides were completely dried using a SpeedVac centrifuge at 30 °C, suspended in 2% ACN and 0.,1% formic acid and analyzed by a nano-UHPLC-MS/MS system using an Ultimate 3000 RSLC coupled to an Orbitrap Fusion Tribrid mass spectrometer (Thermo Scientific Instrument).

Elution was performed with an EASY spray column (75 μm × 50 cm, 2 μm particle size, Thermo Scientific) at a flow rate of 250 nl/min with a 150 min non-linear gradient consisting of 8 min wash with 2% buffer B (80% ACN, 20% H_2_O, 5% DMSO and 0.1% FA), then increasing to 30% B over 97 min, with a further increase to 50% B in 20 min, followed by a 5 min wash at 80% B and a 20 min re-equilibration at 2% B. MS scans were acquired at a resolution of 120,000 between 375 and 1500 m/z and an AGC target of 4.0E5. MS/MS spectra were acquired in the linear ion trap (rapid scan mode) after collision induced dissociation (CID) fragmentation at a collision energy of 35% and an AGC target of 4.0E3 for up to 250 ms. For precursor selection, were prioritized the least abundant signals. Ions with 2 m/z were scheduled for CID/IT analysis with the same parameters applied as above. Charge states 3–7 with minimum precursor intensity of 500,000 were scheduled for analysis by a fast HCD/FT scan of maximal 40 ms at a resolution of 15,000. The remaining charge state 3–7 ions with maximum intensity of 500,000 were scheduled for analysis by CID/IT as described above. Dynamic Exclusion was set at 30 s.

MaxQuant software version 1.6.2.6 was used to process data. A false discovery rate was set at 0.01 for the identification of proteins, peptides and peptide-spectrum match (PSM). A minimum of 6 amino acids was required for peptide identification. Andromeda engine, incorporated into MaxQuant software, was used to search MS/MS spectra against Uniprot human database (release UP000005640_9606 April 2018). In the processing the variable modifications are Acetyl (Protein N-Term), Oxidation (M), Deamidation (NQ), by contrast, the Carbamidomethyl (C) was selected as fixed modification.

The mass spectrometry data have been deposited to the ProteomeXchange Consortium via the PRIDE partner repository^67^ with the dataset identifiers:

Project accession: PXD022512; Reviewer account details: 

Username: reviewer_pxd022512@ebi.ac.uk.

Password: ugq1d5WA.

### ELISA assay

To quantify TATA-binding protein-associated factor 15 (TAF15) S100 protein B (S100B), Thymosin beta-4 (TMSB4X), CD109, 14.3.3 and HSP90 alpha proteins in CSF ELISA kit were used, purchased from MyBiosource (MBS9316981, San Diego, USA), Cloud-Clone Corp (SEA56THu, Houston, USA), Cloud-Clone Corp (CEB609Hu, Houston, USA), Cloud-Clone Corp (SEB458Hu, Houston, USA), CUSABIO (CSB-EL026288HU, Wuhan, China) and StressMarq Biosciences (SKT-107-–96, Victoria , Canada), respectively. Each kit was performed following the manufacturer instructions. Samples were diluted 1:50 in the solution provided by each kit. Each assay of either standard or sample was conducted in triplicate and a box plot was used to visualize the difference in protein levels. In the box plots, each circle corresponds to the mean of the technical triplicate of each sample. The lower detection limit of each assay was determined as the lowest protein concentration that could be differentiated from blank.

### Statistical analysis

Statistical analysis was conducted as reported previously^47^. Mass spectrometry dataset was filtered (70% identity in at least one group) normalized and analyzed by unsupervised hierarchical clustering using multidimensional scaling (MDS) with k-means and Spearman’s Correlation. ANOVA test was used to identify the proteins statistically changing between control and the three tumor class groups i.e. low-grade gliomas and glyoneuronal tumors (LGG and GT), embryonal tumors (EMB) and other brain tumors. By contrast, the statistical difference among control and brain tumor groups, or between LGG plus GT/pilocytic astrocytoma or EMB/MB and all other brain tumors, was assessed using T-test. Besides, in order to establish the priority and relevance of the identified proteins, as well as to further reduce the choice of the proteins highlighted by the statistical analysis, we utilized two other method of analysis i.e. Partial Least Square Discriminant analysis (PLS-DA) and a non-linear Support Vector Machine (SVM). In particular, priority was determined using the rank list and variable importance in projection (VIP) score, resulting respectively from SVM and PLS-DA analyses. In ANOVA and T -test, proteins were considered significantly differentially expressed with 70% of identity in at least one group, power of 80% and adjusted P -value ≤ 0.05 after correction for multiple interactions (Benjamini–Hochberg). In addition for T test, a fold change ≥ 2 was necessary. Volcano plot was used to quickly visualize the statistical differences and the cutoff lines for the adjusted P value ≤ 0.05 and fold change ≥ 2 time were established using the function y = c/(x − x_0_)^68^. SVM is a non-probabilistic machine-learning method of binary classification/prediction proposed by Vapnik^69^. In SVM learning, ANOVA test was utilized to optimize the feature selection. The fourfold cross-validation approach was applied to estimate prediction and classification accuracy. Matrix was randomly divided into two parts: one for learning (65%) and another one (35%) to test prediction accuracy. Learning was repeated until all possible subject combinations in the two groups were done. The resulting core panel of proteins was uploaded in Cytoscape software, and various apps: i.e. Enrichment Map, ClusterMaker2 and AutoAnnotate, were used to construct a protein–protein interaction network to identify the principal biological processes and pathways involved. Gene Ontology (GO) annotations were extracted from the Gene Ontology Consortium (http://www.geneontology.org/). Heatmap diagrams were used to visualize the differences in protein intensity, after Z score normalization. In particular, the intensity values above, below or equal to the mean value are depicted as positive (red), negative (blue) or equal to zero (white), respectively.

For the ELISA assay, the Kruskal–Wallis test for unpaired samples was used to assess the difference in the levels of the potential biomarkers among each clinical group. Results are expressed as medians and interquartile range (IQr). A value of P ≤ 0.05 after Dunn’s correction for multiple comparison was considered statistically significant. Receiver operating characteristic (ROC) curves were generated to assess the diagnostic efficiency of each assay. AUC values were classified as: 0.5, not discriminant; 0.5–0.6, fail; 0.6–0.7, poor; 0.7–0.8, fair; 0.8–0.9, good and 0.9–1, excellent. Youden's index and Likelihood ratio were used to identify the cutoff and the diagnostic performance of each assay, respectively. Statistical analysis was performed using OriginLab Pro and the latest version of software package *R* available at the time of the experiments.

### Ethics statement

The study design was done following the guidelines of the local Ethics Committee that approved the study (n. 18 of 31 october 2013, protocol n. 176, Ethic committee of “G. d’Annunzio” University and ASL N.2 Lanciano-Vasto-Chieti, Italy). All subjects and/or their legal Guardians were informed about the procedures and provided written informed consent to participate in the study. In order to protect human subject identity, a code number was employed for specimen identification.

## Supplementary Information

Supplementary Information 1.

Supplementary Information 2.
