# Supplementary material for: Potential biomarkers of childhood brain tumor identified by proteomics of cerebrospinal fluid from extraventricular drainage (EVD)
Source: Sci Rep. 2021 Jan 19;11:1818. doi: 10.1038/s41598-020-80647-w (PMC7815722; doi:10.1038/s41598-020-80647-w)

**Supporting Information**

**POTENTIAL BIOMARKERS OF CHILDHOOD BRAIN TUMOR IDENTIFIED BY PROTEOMICS OF CEREBROSPINAL FLUID FROM EXTRAVENTRICULAR DRAINAGE (EVD)**

Maurizio Bruschi^1*^, Andrea Petretto^2*^,Armando Cama^3^,Marco Pavanello^3^,Martina Bartolucci^2^,Giovanni Morana^4^,Luca Antonio Ramenghi^5^, Maria Luisa Garré^6^, Gian Marco Ghiggeri^7^,Isabella Panfoli^8#†^and Giovanni Candiano^1#^

*^1^Laboratory of MolecularNephrology, IRCCS Istituto Giannina Gaslini, Genoa, Italy.*

*^2^ Core Facilities –ClinicalProteomics and Metabolomics, IRCCS Istituto Giannina Gaslini, Genoa, Italy.*

*^3^Department of Neurosurgery, IRCCS Istituto Giannina Gaslini, Genoa, Italy.*

*^4^Unit of Neuroradiology, IRCCS Istituto Giannina Gaslini, Genoa, Italy.*

*^5^Neonatal Intensive Care Unit, IRCCS Istituto Giannina Gaslini, Genoa, Italy.*

*^6^Department of Neuroncoloy, IRCCS Istituto Giannina Gaslini, Genoa, Italy*

*^7^UO of Nephrology, Dialysis andTransplantation, IRCCS IstitutoGianninaGaslini, Genoa, Italy.*

*^8^ Dipartimento di Farmacia (DIFAR), Università di Genova, Genoa, Italy.*

*These authors equally contributed as first Author

# These authors equally contributed as last Author

^†^Corresponding Author:

Prof. Isabella Panfoli

Università di Genova-DIFAR

V.le Benedetto XV,3

16132 Genova, Italy

phone: +39 010 353.7397

fax: +39 010 353.8153

e-mail: [panfoli@difar.unige.it](mailto:panfoli@difar.unige.it)

**Supporting Table 1. List of all proteins identified in the child's cerebrospinal fluid from drainage samples.** The symbol "+" indicates the proteins presences in each groups and those filtered for 70% of identity in at least one groups**.**

**Supporting Table 2. List of 741 proteins identified in the child's cerebrospinal fluid from drainage filtered for 70% of identity in at least one groups.** The symbol "+" indicates the statistically significant changed proteins or those previously described as associated with brain tumors (www.uniprot.org). Mean of Log2 of Label-Free Quantification Intensity, standard deviation and the results of the statistical analysis are reported for each groups.

**Supporting Table 3. List of 104 proteins that maximize the discrimination between Control, LGG and GT, EMB and Other brain Tumor samples.** Mean of Log2 of Label-Free Quantification Intensity and their standard deviation are reported for each groups. Besides, results of PLS-DA (VIP score) and SVM (rank) are reported for each protein. In Both analysis the proteins in the first positions are the most promising biomarkers to distinguish control from brain tumor and stratify this last groups in LGG and GT, EMB and other Brain Tumor.

**Supporting figure 1. Volcano plots of CSF proteome from EVD of pilocytic astrocytoma and medulloblastoma compared with all other classes of brain tumor.** Volcano plot for **A)** PA or **B)** MB compared to all other brain tumors. Grey, open black, open red and red circles indicate respectively the changes in non-significant, significant and previously described in brain tumor or in pilocytic astrocytoma/medulloblastoma proteins among the two conditions. Black line indicates the limits of statistically significant.

**Supporting Figure 2. Enrichment of biological process**. Diagram of enrichment analysis of biological process mapped as a network. Nodes (circles) and edges (line) represent respectively the biological process and their interconnection. Node color intensity (from light to dark) and size are in accordance to the enrichment in the control (blue) or brain tumor (red) conditions and their p-value, respectively. The biological processes are grouped in four clusters (light yellow ellipses) in function of their GO annotation.

**Supporting Figure 3. Received operating characteristic (ROC) curves analysis of each ELISA assay.** ROC curve analysis for A) S100B, B) TAF15, C) TMSB4X, D) CD109, E) 14.3.3 and HSP90 alpha proteins. Each ELISA resulted excellent (AUC>0.9). See Table 2 for the detail.


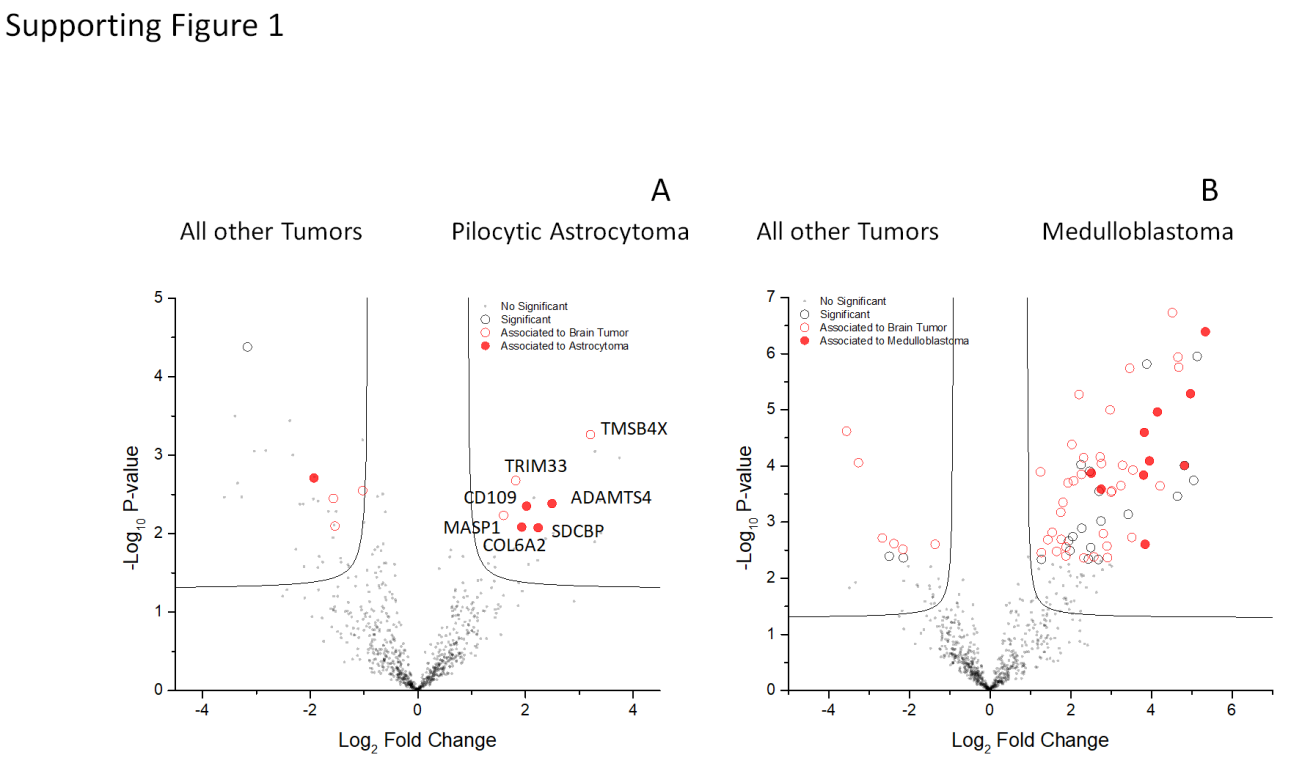


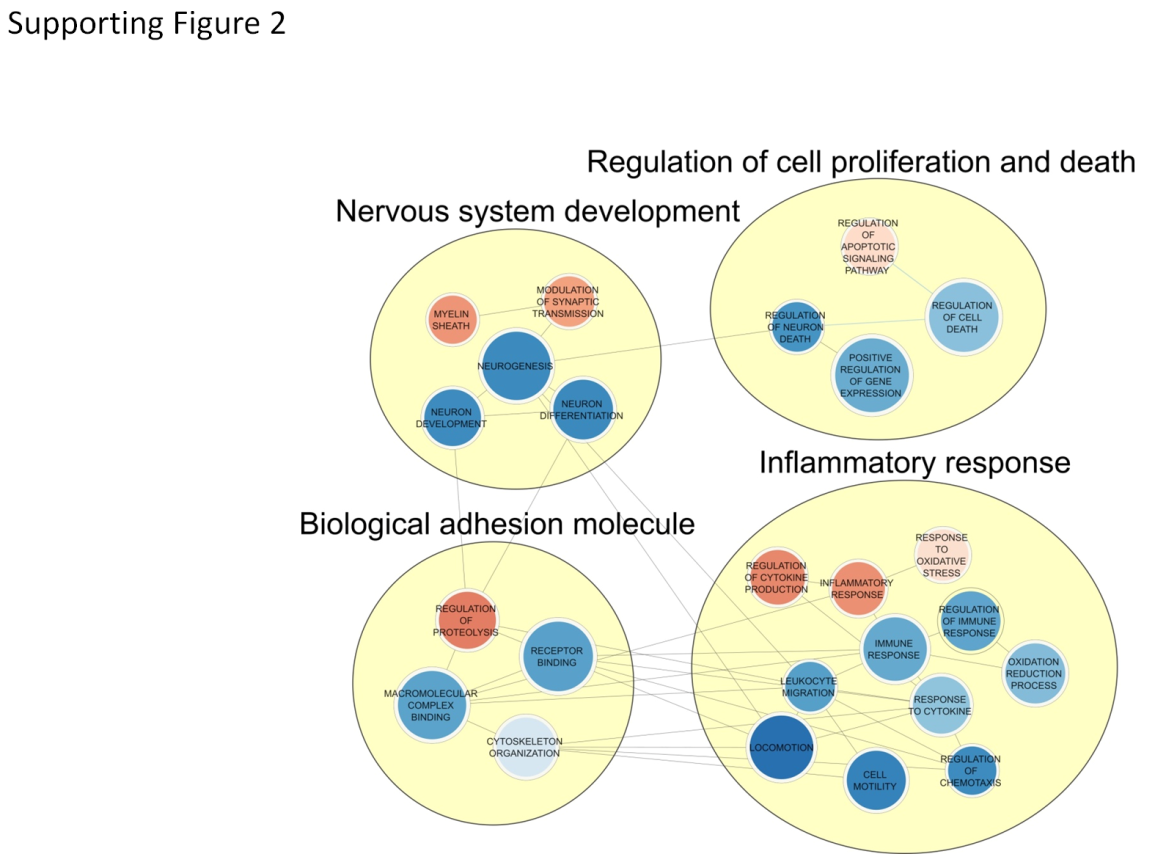


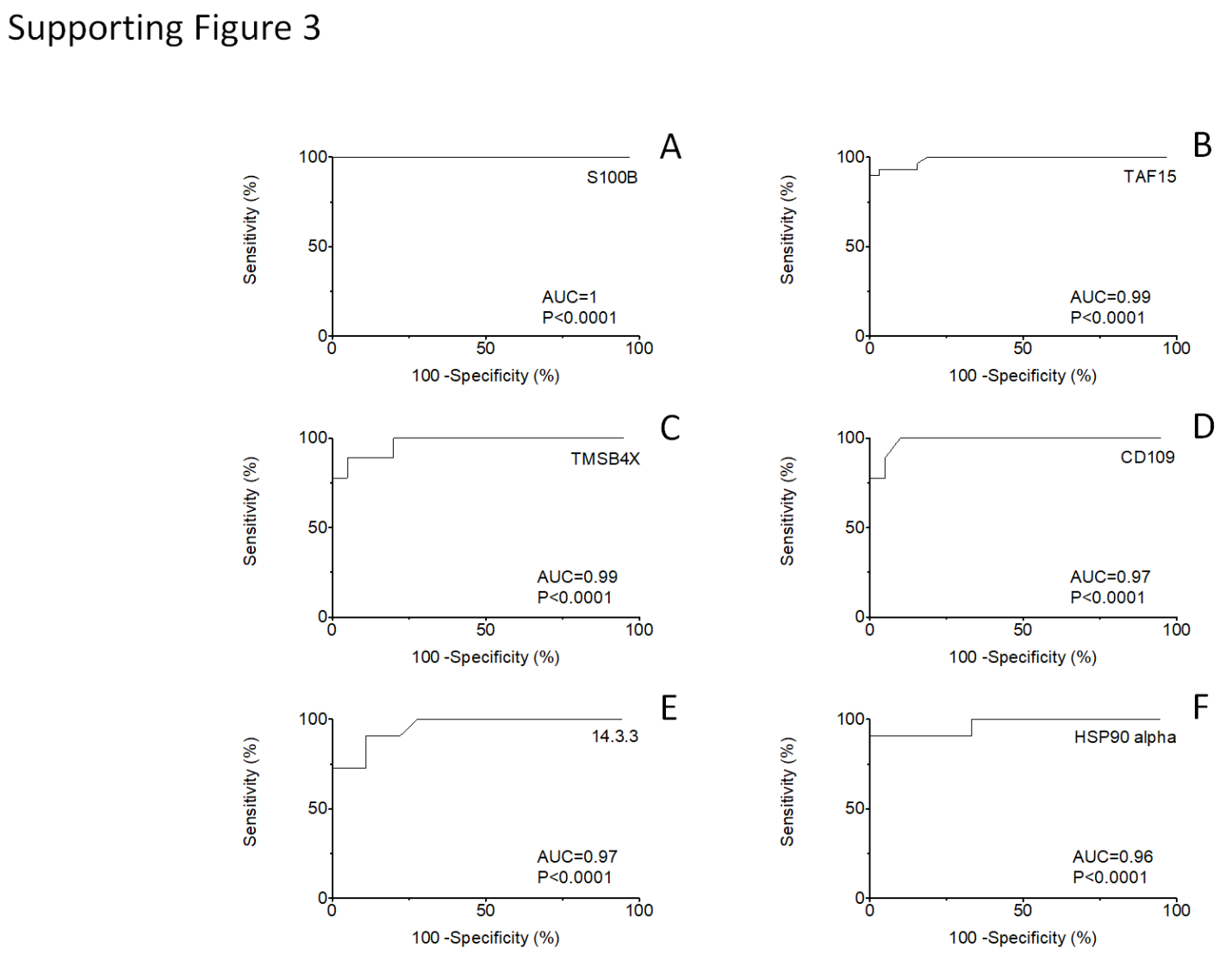

Supplement: Supplementary file 1 — Supplementary Information 1. [file 41598_2020_80647_MOESM1_ESM.docx]
